# Usefulness of a finger-mounted tissue oximeter with near-infrared spectroscopy for evaluating the intestinal oxygenation and viability in rats

**DOI:** 10.1007/s00595-020-02171-8

**Published:** 2020-10-27

**Authors:** Yuhi Suzuki, Masayoshi Yamamoto, Kosuke Sugiyama, Toshiya Akai, Katsunori Suzuki, Takafumi Kawamura, Mayu Sakata, Yoshifumi Morita, Hirotoshi Kikuchi, Yoshihiro Hiramatsu, Kiyotaka Kurachi, Naoki Unno, Hiroya Takeuchi

**Affiliations:** 1 Second Department of Surgery, Hamamatsu University School of Medicine, 1-20-1 Handayama, Hamamatsu, Shizuoka 431-3192 Japan; 2 Division of Vascular Surgery, Hamamatsu Medical Center, 328 Tomitsuka, Hamamatsu, Shizuoka 432-8580 Japan

**Keywords:** Rat, Intestine, Ischemia reperfusion, Near-infrared spectroscopy

## Abstract

**Purpose:**

To investigate the utility of the device for evaluating intestinal oxygenation and viability using an animal model.

**Methods:**

Sprague–Dawley rats underwent laparotomy under general anesthesia, and the blood vessels in the terminal ileum were clamped to create ischemia. We measured the regional tissue oxygenation saturation (rSO_2_) using an oximeter after 1, 3, and 6 h of vessel clamping. Ischemic tissue damage was assessed using a histological score. The intestine was reperfused after each clamping period, and intestinal rSO_2_ and survival rate were evaluated.

**Results:**

When reperfusion was performed at 1 and 3 h after ischemia, rSO_2_ increased after 10 min, and it improved to the same level as for normal intestine after 1 h; all rats survived for 1 week. In contrast, after 6 h of ischemia, rSO_2_ did not increase after reperfusion, and all animals died within 2 days. The histological scores increased after 1 h of reperfusion, with longer clamping periods.

**Conclusion:**

A finger-mounted tissue oximeter could evaluate intestinal ischemia and the viability, which is thus considered to be a promising result for future clinical application.

## Introduction

Intestinal ischemia caused by a strangulated ileus and non-occlusive mesenteric ischemia is frequently seen in gastrointestinal surgery. Most closed-loop ileus cases are associated with intestinal ischemia, and once the ischemia becomes irreversible, the intestine cannot be preserved even if the strangulation is released [1–3]. Intraoperative decisions must be prompt and accurate to determine whether the intestine can be preserved. However, currently, most surgeons rely on personal experience to make these decisions, and new monitoring devices that can provide objective evaluations are needed. Currently, intestinal fluorescence imaging using indocyanine green (ICG) is widely used clinically to objectively evaluate intestinal ischemia. However, problems with this technique include its complexity, quantitativity, and repeatability [4, 5].

A finger-mounted tissue oximeter (Toccare; Astem Co., Ltd., Kawasaki, Japan) is a newly developed device for measuring rSO_2_ using near-infrared spectroscopy (NIRS). This device enables iterative and quantitative rSO_2_ evaluation of the target tissue/organ using a simple method and has been reported to be useful in obstetrics and gynecology, vascular surgery, and plastic surgery [6–11]. If this device can be used to evaluate intestinal ischemia, we can determine whether the intestine can be preserved, intraoperatively, using a rapid and quantitative evaluation. In this study, we evaluated the usefulness of a finger-mounted tissue oximeter to assess intestinal ischemia, in an animal model.

## Materials and methods

### Animals

Male Sprague–Dawley rats (400–550 g, *n* = 128) from Japan SLC (Hamamatsu, Japan) were maintained under a 12/12-h light/dark cycle with constant conditions of room temperature and air humidity. Prior to the experiment, all rats were allowed free access to a standard diet and water.

This study was approved by the Institutional Animal Care and Use Committee of Hamamatsu University School of Medicine (Permission number: 2017090). All experimental procedures were conducted in accordance with the Fundamental Guidelines for Proper Conduct of Animal Experiment and Related Activities in Academic Research Institutions (Ministry of Education, Culture, Sports, Science and Technology of Japan) and the National Institutes of Health Guide concerning the Care and Use of Laboratory Animals.

### Experimental groups

Three groups of rats were used in this study:

- Control group (*n* = 23): laparotomy was performed without clamping. Control groups were used in the experiments for ischemic intestine (*n* = 4) and ischemia/reperfusion (I/R) injured intestine (*n* = 19).
- Clamp group (*n* = 38): the blood flow of intestine was clamped. This group was sub-dived into two groups. Each group was subjected to ischemic injury for either 1 h (Clamp 1 h group; *n* = 28) or 6 h (Clamp 6 h group; *n* = 10).
- I/R group (*n* = 67): ischemic manipulation as previously described was performed for each appropriate period. After the clamp was released, 1 h of reperfusion was applied. This group was sub-divided into three groups. Each group was subjected to I/R injury for 1 h (1 h I/R group; *n* = 24), 3 h (3 h I/R group; *n* = 23) and 6 h (6 h I/R group; *n* = 20). In the survival experiment, five rats from each group were used. rSO_2_ and mortality was compared between 1 and 3 h I/R group versus 6 h I/R group.

### Experimental details of the ischemia/reperfusion models

The experimental procedures were based on those described in previous studies [12–14]. Briefly, the rats were intraperitoneally anesthetized with 0.4 mg/kg medetomidine, 2.0 mg/kg midazolam, and 5.0 mg/kg butorphanol, and injections were repeated every 2 h to maintain anesthesia. Under anesthesia, laparotomy was performed through a ventral midline abdominal incision. The blood vessels in the terminal ileum were clamped using bulldog forceps (C-42-S-2; Natsume Seisakusho Co., Ltd., Tokyo, Japan) for specific lengths of time. For the reperfusion evaluation, clamping forceps were released at specific time points to restore blood flow. In the survival experiment, animals were monitored twice a day for 1 week after operation regarding general appearance, viability, morbidity, and behavior of stools. Coma, effort respiration, heavy vomit, hematemesis, diarrhea, and melena were defined for humane endpoint. If any of the symptoms were observed, then the rats were euthanized within 1 h of onset.

We euthanized all rats with 200 mg/kg pentobarbital sodium after each experiment. All efforts were made to protect the animals and minimize their suffering during the study.

### Measuring intestinal rSO_2_

rSO_2_ was measured by gently placing the finger-mounted tissue oximeter on the surface of the terminal ileum until the value stabilized after a few seconds, and the value was recorded manually. During measurement, a 5-mm-thick black rubber plate was placed on the dorsal intestine to prevent near-infrared scattering. The oximeter and rubber plate were covered with an echo probe cover (CIV-Flex™ Transductal Cover; Civco Medical Solutions, Orange City, IA) to maintain an aseptic surgical field.

### ICG angiography

After clamping the intestine to stop the blood flow for 1 h, 0.075 mg of ICG (Daiichi Sankyo Co., Ltd., Tokyo, Japan) was injected through the right femoral vein. After 20 s, the surface of the intestine was observed using an NIR camera system (Photodynamic Eye; Hamamatsu Photonics K.K., Hamamatsu, Japan), which activates ICG with emitted light (wavelength, 760 nm) and filters out light with a wavelength below 820 nm. The light source to detect ICG consisted of 760-nm light-emitting diodes, and the detector was a charge-coupled device camera. Fluorescence intensity was quantified using the ImageJ software program (National Institutes of Health, Bethesda, MD) as previously described [15].

### Histological analysis

After I/R manipulation, the terminal ileum was excised and fixed with 10% formalin overnight at room temperature, then embedded in paraffin wax. Formalin-fixed and paraffin-embedded slides were counterstained with hematoxylin and eosin, and histopathological grading was determined using the Park/Chiu score as follows: 0 = normal mucosa; 1 = subepithelial space at the tips of the villi; 2 = extension of subepithelial space with moderate lifting; 3 = massive lifting down the sides of the villi, with some denuded tips; 4 = denuded villi, dilated capillaries; 5 = disintegration of the lamina propria; 6 = crypt layer injury; 7 = transmucosal infarction; and 8 = transmural infarction [16–19]. The histopathological score was evaluated independently by three observers. A median score for all sections was evaluated.

### Statistical analysis

All statistical analyses were performed using the EZR version 1.41 software program (Saitama Medical Center, Jichi Medical University, Saitama, Japan), which is a graphical user interface for R (The R Foundation for Statistical Computing, Vienna, Austria). More precisely, EZR is a modified version of R commander designed to add statistical functions frequently used in biostatistics [20]. Before analyzing either rSO_2_ or fluorescence intensity, we used the Shapiro–Wilk test to confirm normality of the distribution. The paired t-test was used for compatible conditions. For multiple comparisons, two-way analysis of variance (ANOVA) or one-way repeated ANOVA and Holm’s test for post hoc were used to compare intestinal rSO_2_ or fluorescence intensity. Evaluating Spearman's rank correlation analysis was performed to analyze the interrelation between the logarithms of rSO_2_ and period of intestinal ischemia. The Kruskal–Wallis test and Steel–Dwass test were used to compare Park/Chiu scores. All results except for histopathological scoring and survival analysis were presented as the mean ± standard error of the mean. Park/Chiu score was presented with a dot plot. For the survival analysis, the Kaplan–Meier method was used and differences were compared by the log rank test. For each test, *p*-values < 0.05 were considered to be significant.

## Results

### Evaluation of ischemia by intestinal vessel clamping

**Fig. 1:**
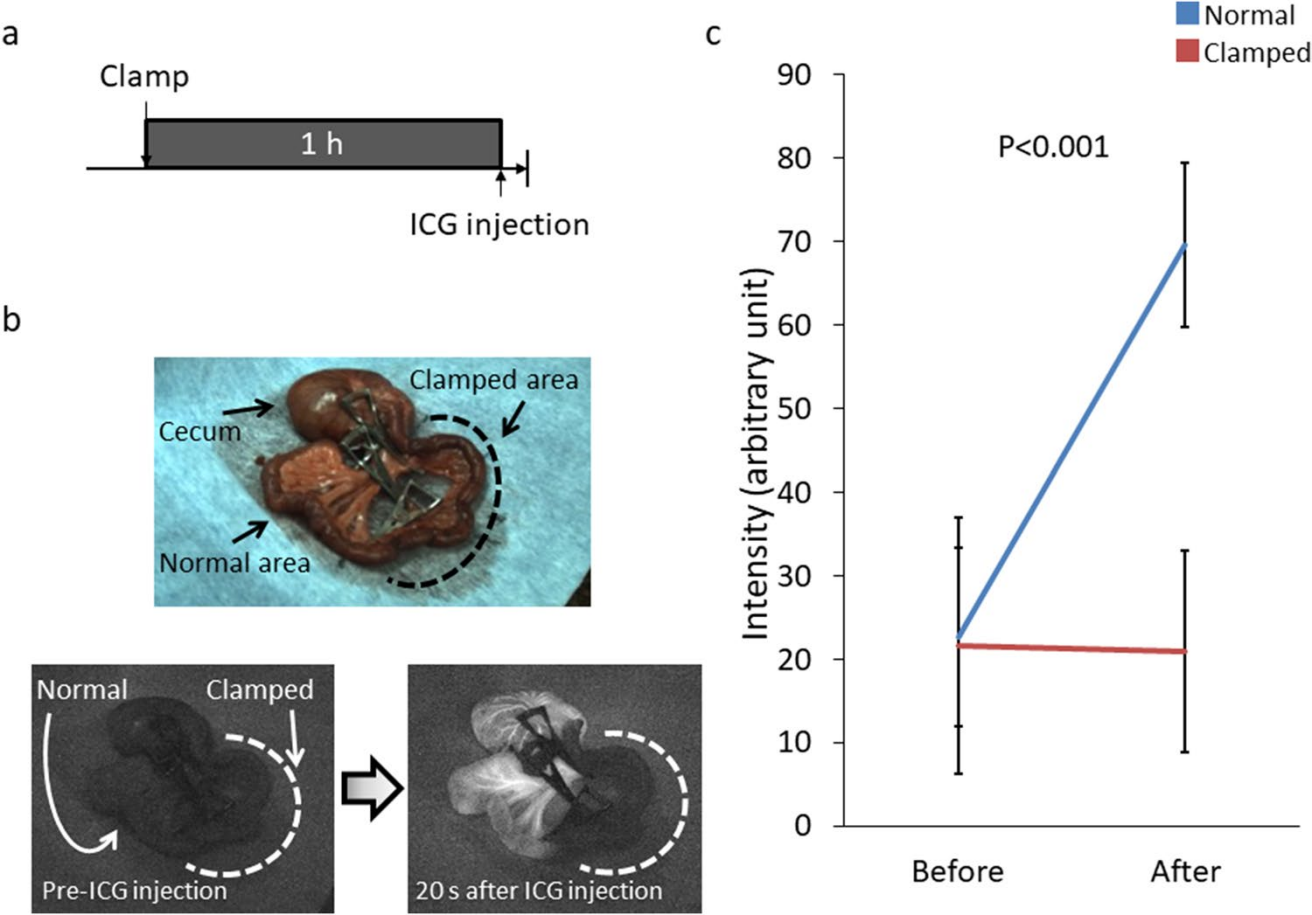
Evaluation of intestinal ischemia in an animal model by using ICG fluorescence imaging. **a** Experimental design of the ICG fluorescence. **b** Evaluation of ischemia by ICG fluorescence. A macroscopic view of the normal/clamped intestine. Upper image: under white light. Lower image: fluorescent image before (left) and 20 s after ICG injection (right). **c** Quantification of fluorescence intensity using the ImageJ software program (*n* = 10). *s* second; *ICG* indocyanine green

**Fig. 2:**
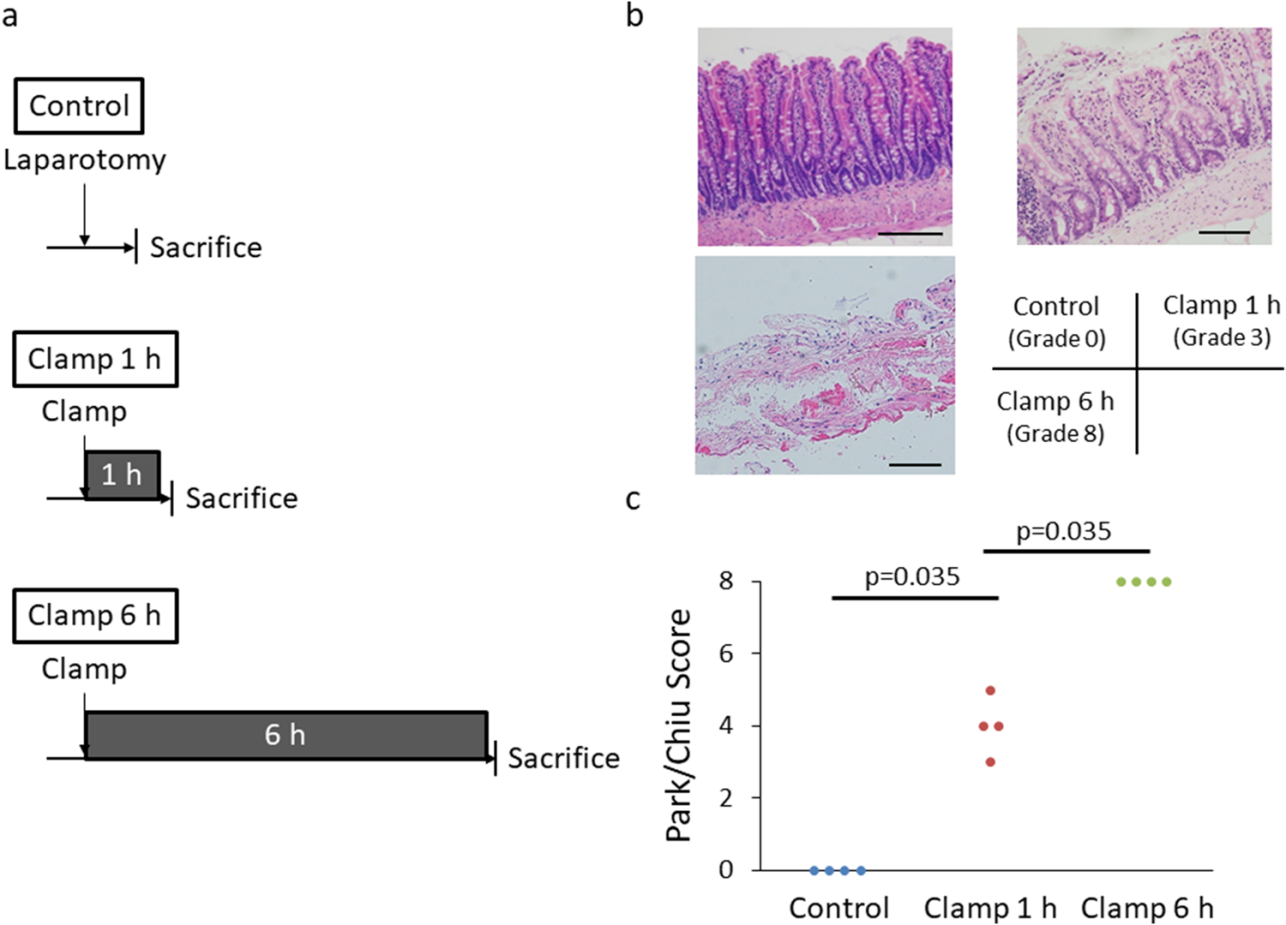
Evaluation of intestinal ischemia in an animal model by Park/Chiu histological scoring. **a** Experimental design of Park/Chiu scoring. **b** A representative microscopic view of intestinal tissue after the indicated duration of vessel clamping. Upper image: Normal intestinal mucosa (left) and denuded tip of villus (right). Lower image: Necrosis of mucosa and muscularis propria. **c** Histological quantification of tissue ischemia according to the Park/Chiu scoring system (*n* = 4 per group). Scale bar, 100 µm. *h* hour

We first created a model of intestinal ischemia by clamping the vessels of the terminal ileum and evaluated the ischemia by ICG fluorescence and a histological analysis. ICG fluorescence of the terminal ileum showed no ICG fluorescence in the area after 1 h of vessel clamping (Fig. 1a, b). The fluorescence intensity of the clamped area was significantly lower than that for normal intestine (Fig. 1c). Histologically, intestinal tissue showed ischemic damage limited to the villi after 1 h of vessel clamping, and these findings extended into the seromuscular layer after 6 h of clamping (Fig. 2a). A quantitative analysis by Park/Chiu score showed a significant increase in scores over time (Fig. 2b, c). These results indicated that the clamped area was indeed ischemic, in this model.

### Evaluation of rSO_2_ in the ischemic intestine

**Fig. 3:**
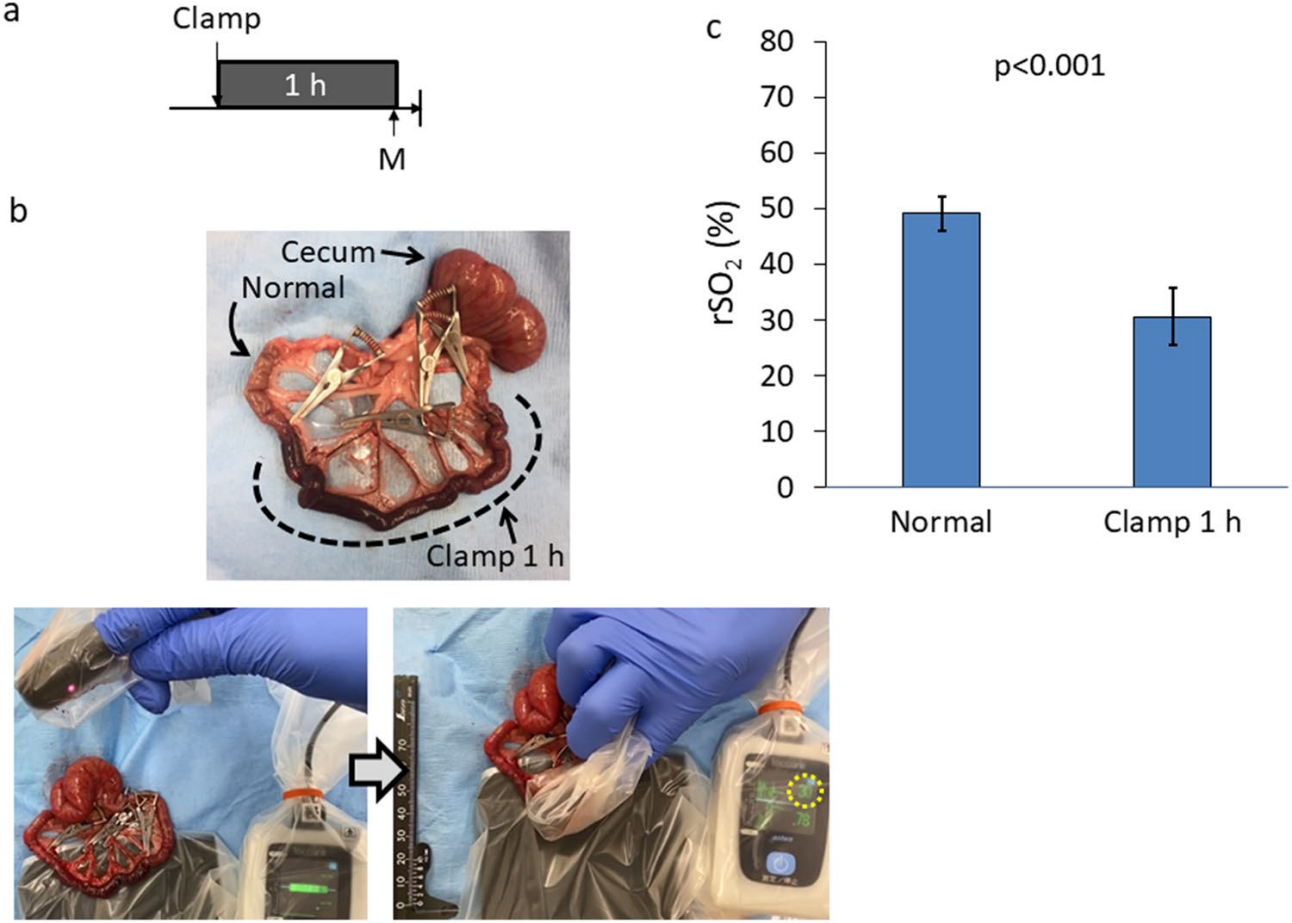
rSO_2_ measurement of the intestine. **a** Experimental design of rSO_2_ measurement of 1-h clamped intestine. **b** Representative images of 1-h clamped intestine and intestinal rSO_2_ measurement. Upper image: Intestine of clamped region was more reddish brown than normal intestine. Lower image: Intestinal rSO_2_ was measured under “finger-mounted” state. The value in the dotted circle indicates rSO_2_. **c** rSO_2_ for normal and ischemic intestine after 1 h of vessel clamping (*n* = 14). *h* hour; rSO_2_ regional tissue oxygenation saturation; *M* measurement of rSO_2_

**Fig. 4:**
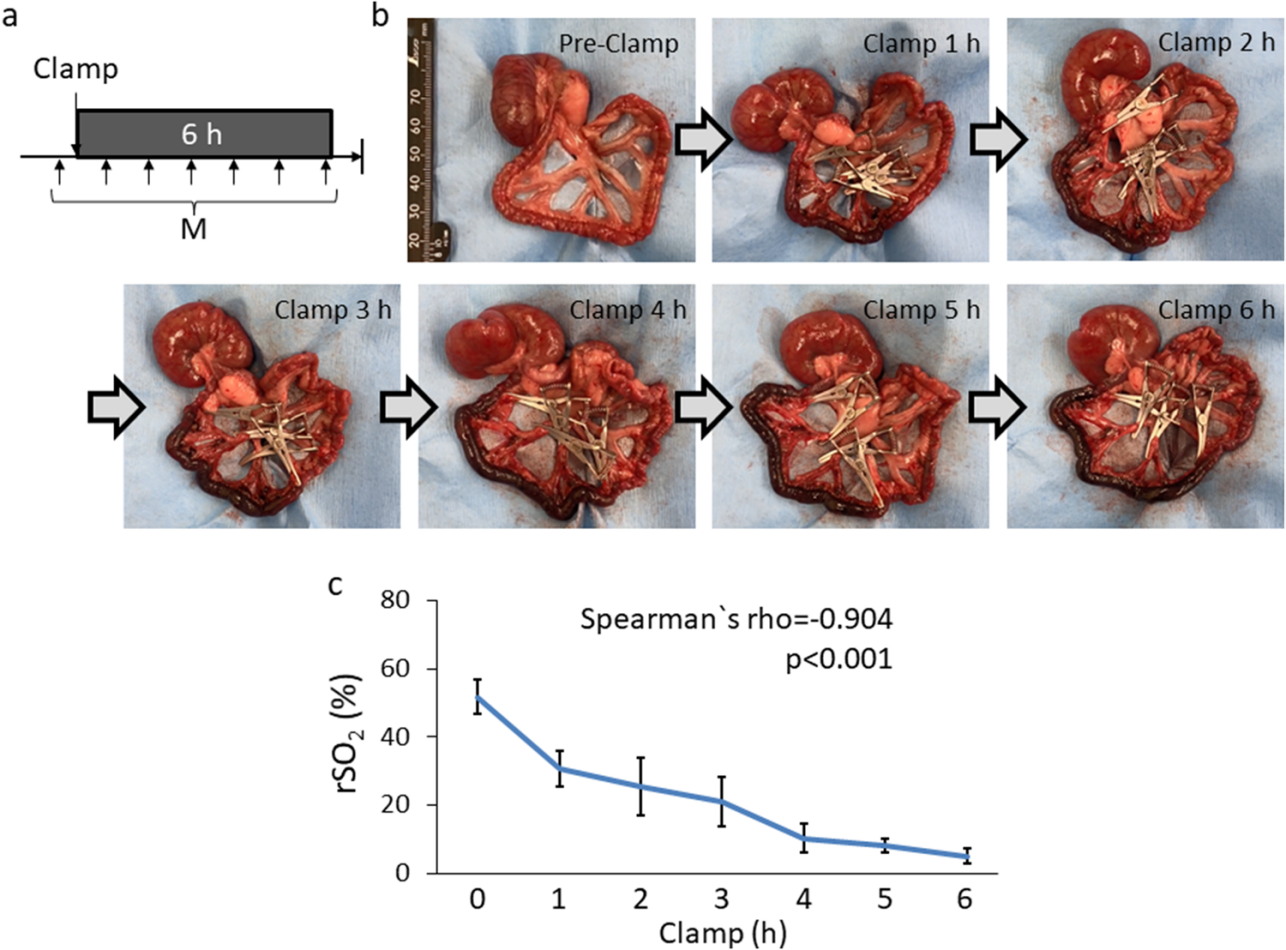
rSO_2_ measurement of the intestine hourly. **a** Experimental design of intestinal rSO_2_ hourly measurement during 6-h clamping. **b** Representable hourly images of intestine during 6 h clamping the blood flow. All sequential images were taken from the same rat. (c) Changes in rSO_2_ every hour up to 6 h of clamping and secondary ischemia (n = 6). *h* hour; *rSO*_*2*_ regional tissue oxygenation saturation; *M* measurement of rSO_2_

To verify the usefulness of the finger-mounted tissue oximeter to assess intestinal ischemia, we evaluated intestinal rSO_2_ after 1 h of vessel clamping (Fig. 3a). As shown in Figs. 3b, c, the rSO_2_ of the ischemic area was lower than that for the normal intestine. In order to verify the sequential rSO_2_ change, rSO_2_ values were tracked hourly for 6 h (Fig. 4a). During the clamping of the blood flow for 6 h, the macroscopical appearance of the intestine and mesentery showed ischemic and congestive change characterized by reddish color change. The changes that derived from ischemia and congestion changed from mild to severe over time. Eventually the intestine became dark red and mesentery became blood red with hematoma (Fig. 4b). Along with the changes in the macroscopical appearance, intestinal rSO_2_ was decreased significantly over time (Fig. 4c).

### rSO_2_ after I/R

**Fig. 5:**
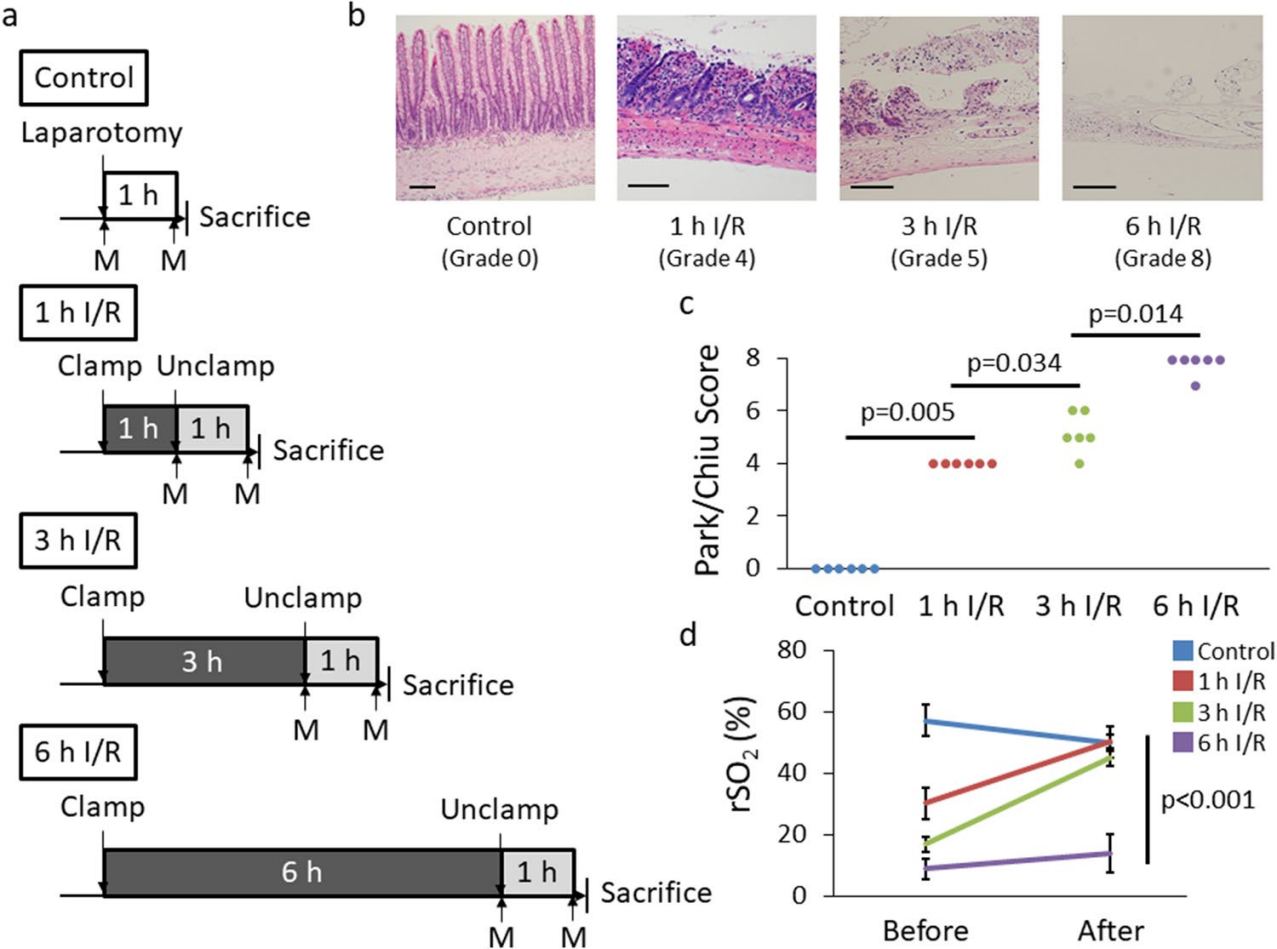
Intestine after I/R injury. **a** Experimental design of I/R injury among four groups. **b** Representative microscopic images of intestinal tissue after the indicated duration of I/R. Intestinal tissue which was not imposed any injury (left). Villi were denuded by I/R injury (second from the left). Villus tissue was lost but lamina propria did not necrotize (second from the right). Mucosa and muscularis propria became necrosis (right). **c** Histological quantification of tissue damage according to the Park/Chiu scoring system after the indicated duration of I/R (*n* = 6 per group). **d** rSO_2_ before and 1 h after reperfusion (*n* = 4 per group). Scale bar, 100 µm. *I/R* ischemia/reperfusion; *rSO*_*2*_ regional tissue oxygenation saturation; *M* measurement of rSO_2_

Next, we assessed intestinal rSO_2_ after I/R (Fig. 5a). When assessing tissue damage by Park/Chiu scores, the score increased as the duration of ischemia increased, reaching a maximum at 6 h (Fig. 5b, c). Next, we assessed intestinal rSO_2_ of 1, 3, and 6-h ischemia followed by reperfusion (1 h I/R, 3 h I/R, 6 h I/R, respectively). After 1 h and 3 h I/R, rSO_2_ recovered to the control level. However, rSO_2_ did not recover after 6 h I/R (Fig. 5d).

**Fig. 6:**
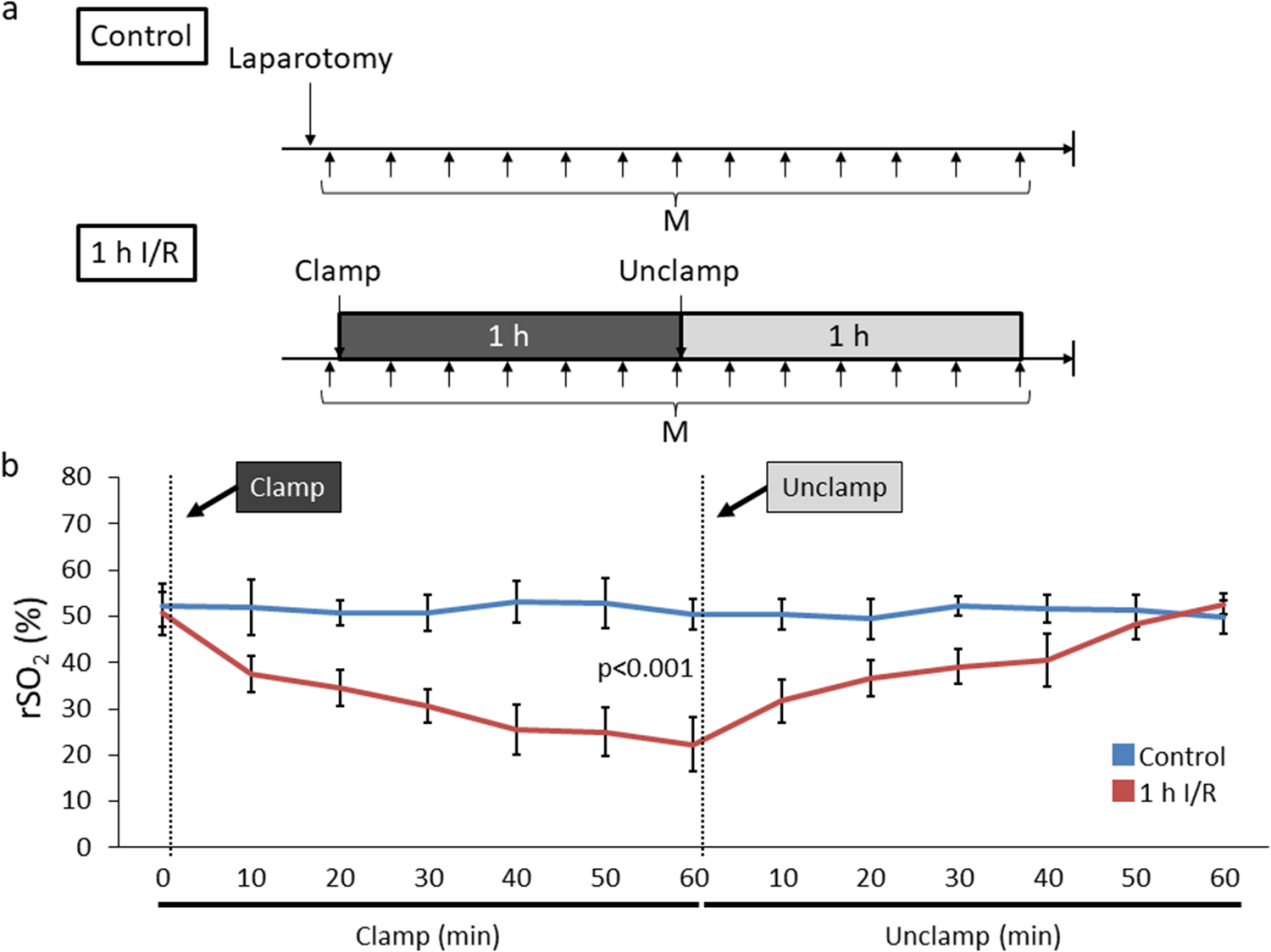
rSO_2_ transition after I/R injury of 1 h I/R. **a** Experimental design of rSO_2_ measurement every 10 min. **b** rSO_2_ changes during 1 h of I/R (*n* = 9 per group). *h* hour; *I/R* ischemia/reperfusion; *min* minute; rSO_2_ regional tissue oxygenation saturation; *M* measurement of rSO_2_

**Fig. 7:**
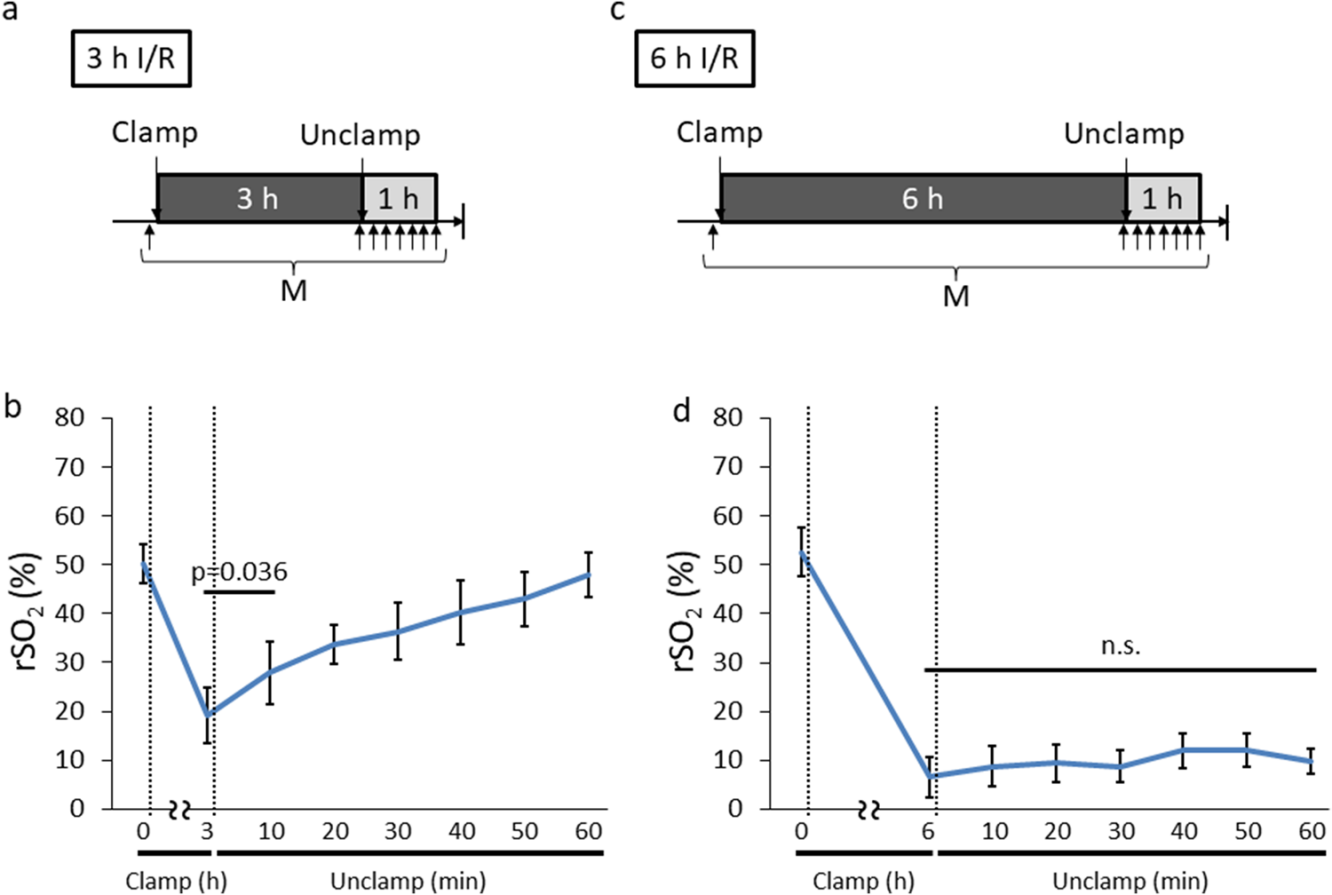
rSO_2_ transition after I/R injury of 3 and 6 h I/R group. **a** Experimental design of rSO_2_ measurement of 3 h I/R group. **b** rSO_2_ changes during 3 h of I/R (*n* = 8). **c** Experimental design of rSO_2_ measurement of 6 h I/R group. **d** rSO_2_ changes during 6 h of I/R (*n* = 5). *h* hour; *I/R* ischemia/reperfusion; *min* minute; *rSO*_*2*_ regional tissue oxygenation saturation; *M* measurement of rSO_2_; *n.s.* not significant

**Fig. 8:**
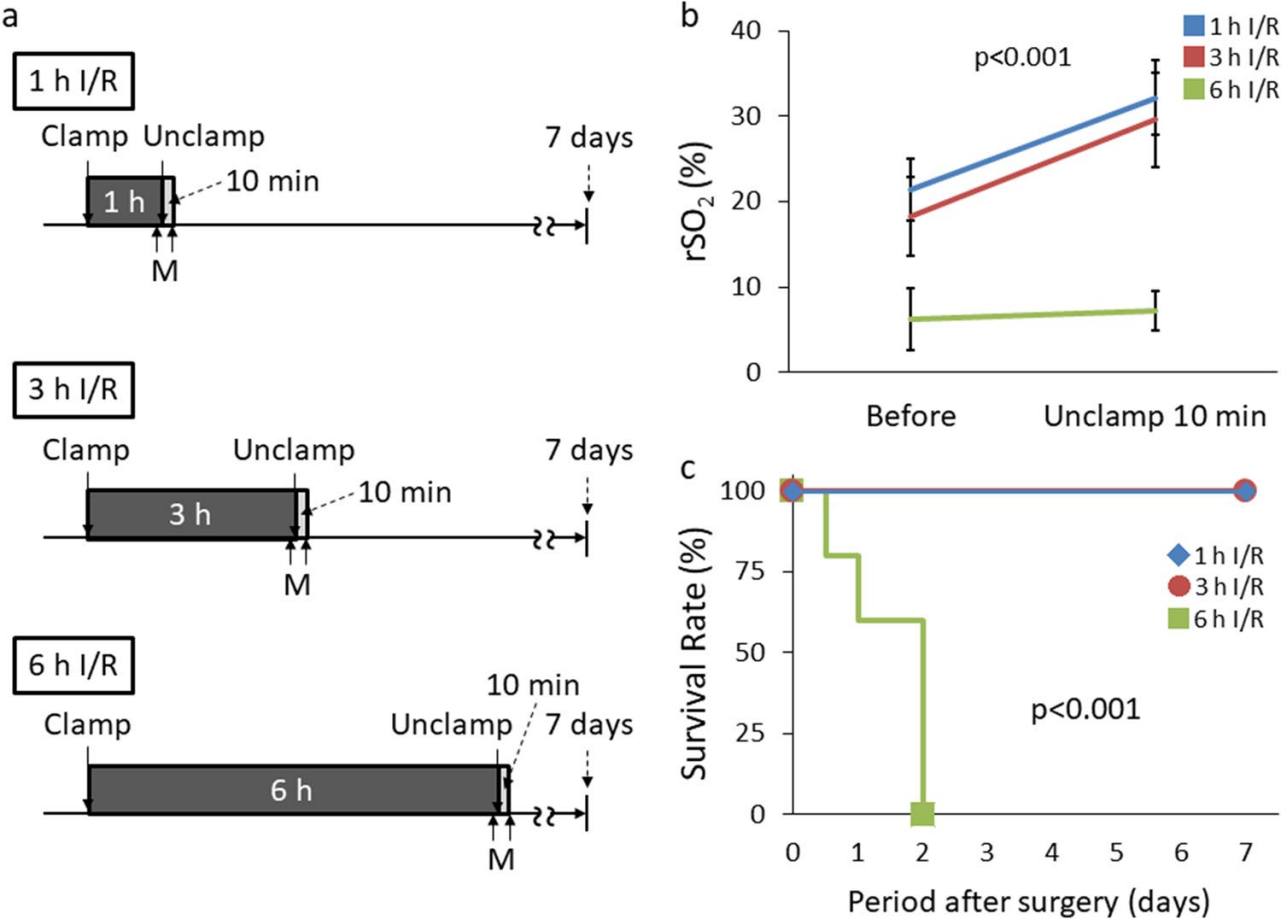
rSO_2_ 10 min after reperfusion and subsequent survival outcomes. **a** Experimental design of rSO_2_ measurement and evaluation of long-term survival. **b** rSO_2_ changes after 10 min of reperfusion during 1, 3 and 6 h I/R (*n* = 5 per group). **c** Representation of the survival for each I/R group (*n* = 5 per group). *h* hour; *min* minute; *rSO*_*2*_ regional tissue oxygenation saturation; *M* measurement of rSO_2_

Next, we assessed the short-term rSO_2_ transitions after I/R injury. We anticipated that if the intestinal ischemia was irreversible, the rats would not survive for 1 week post-procedure. In the 1 h I/R group (Fig. 6a), rSO_2_ decreased gradually during the 1-h vessel clamping. After reperfusion, rSO_2_ increased every 10 min and reached an equivalent level to the control/normal intestine (Fig. 6b), suggesting that the intestine might be salvageable after 1 h of ischemia. After 3 h I/R (Fig. 7a, b), similar to 1 h I/R, rSO_2_ started to increase after 10 min of reperfusion. In contrast, rSO_2_ did not recover after 6 h of ischemia (Fig. 7c, d). Next, we focused on the relationship between variance of rSO_2_ and long-term survival (Fig. 8a). All rats of 1 and 3 h I/R groups in which rSO_2_ was increased after reperfusion survived for 1 week after laparotomy, whereas all five rats of 6 h I/R group in which rSO_2_ was not increased after reperfusion died within 2 days (Fig. 8b, c). Necropsy was performed in all rats which belonged to 6 h I/R group as soon as their death was confirmed. The necropsy findings did not indicate any cause of death other than intestinal necrosis. These results suggest that intestinal ischemia might become irreversible between 3 and 6 h of vessel clamping. In addition, the rSO_2_ of viable and salvageable intestine recovered rapidly after reperfusion.

## Discussion

Currently, intestinal ischemia is evaluated by ICG and Doppler ultrasound, but these methods are associated with problems such as complexity, quantitativity, and repeatability, and no standard method has been established [4, 21–23]. The finger-mounted tissue oximeter used in this study is a simple and quantitative tissue oxygen saturation monitoring device that uses NIRS technology. In this study, we demonstrated the usefulness of this finger-mounted tissue oximeter to evaluate intestinal ischemia for the first time. In addition to analyze intestinal ischemia, we evaluated intestinal viability by comparing the survival period.

NIRS was first reported in the 1980s and it has been used primarily to assess cerebral circulation [23]. Previous NIRS measuring devices were used to evaluate adults’ cerebral blood flow to a depth of 1–3 cm [9, 24, 25], but these devices are unsuitable for assessing intestinal tissue. The thickness of the small intestinal wall is less than 3 or 4 mm, therefore the depth of measurement with previous NIRS devices was considered to be too deep [26, 27]. Recently, the cerebral blood flow of infants or the placental oxygen saturation of fetuses were analyzed using advanced technology of NIRS [28–30]. More recently, a finger-mounted tissue oximeter was developed to measure oxygen saturation of the fetal head, transvaginally [6]. This finger-mounted tissue oximeter has also been used to assess lower limb ischemia in patients with peripheral arterial disease [9, 31].

Measuring rSO_2_ with a finger-mounted tissue oximeter is very simple because the device can be quantified by simply contacting the probe with the target organ. Unlike ICG, these devices provide repeated measurements. The optimize measurement depth is reported to be 4–5 mm from the probe [6, 9]. However, it can be also applied to 3-mm-thick tissue, such as facial skin and subcutaneous tissue of mouse [32]. The rat intestine was approximately 3 mm in thickness, so we used rubber plates behind the intestine in order to avoid penetration and the scattering of light.

We showed that rSO_2_ decreased over time after blood flow interruption. To examine the correlation between the degree of tissue damage and rSO_2_, hourly rSO_2_ and the Park/Chiu score was analyzed up to 6 h. The rSO_2_ decreased over time, and the Park/Chiu score increased over time, suggesting that finger-mounted oximeter evaluates the degree of intestinal ischemia. rSO_2_ was calculated by the concentrations of oxyhemoglobin [O_2_Hb] and deoxyhemoglobin [HHb] in the target tissue as rSO_2_ = [O_2_Hb]/([O_2_Hb] + [HHb]) [6, 11]. We considered the reasons for reducing rSO_2_ by intestinal ischemia to be as follows: the first is the decreasing of oxyhemoglobin by consuming oxygen in the tissue when blood flow is blocked. Interruption of arterial blood flow causes the decrease of oxyhemoglobin supply that results in decrease of oxygen diffusion [16, 17, 33–35]. In our study, injured tissue by I/R indeed showed ischemic change such as microvascular collapse, denuded villi and necrosis. The second is the increase of deoxyhemoglobin by impairment of venous blood flow. The interruption of venous blood flow causes reduction of venous drainage to portal vein. Venous stasis increases tissue pressure and deoxyhemoglobin accumulation that results in hypoxia [16, 17, 35]. Indeed, we found that tissue of I/R injured intestine showed collapse of capillary vessel and venous enlargement.

Furthermore, the rats with recovered rSO_2_ after reperfusion survived for 1 week, and none of the rats with unrecovered rSO_2_ survived. These results suggest that rSO_2_ reflects the viability of the intestinal tract in real time, and that finger-mounted tissue oximeters may be used to determine whether intestine can be preserved intraoperatively. In comparison with 6 h I/R, the tissue damage of 1 h and 3 h I/R were mild. In addition to the results of histological scoring after I/R injury, the survival outcome also clearly reflected the intestinal viability. To determine the actual cause of death of 6 h I/R group as intestinal necrosis is difficult. However, the autopsy of five deceased rats did not suggest other possible causes of death such as intraabdominal hemorrhage or injury of other organs. Therefore, we determined their cause of death as intestinal necrosis of the terminal ileum.

For clinical application, it was necessary to evaluate the viability of the intestine after intraoperative reperfusion and determine whether the intestinal tract can be preserved in a short time. Therefore, we analyzed the intestinal rSO_2_ a short time after reperfusion. Some previous studies of NIRS showed fluctuation of rSO_2_ after reflow of lower limbs by vascular occlusion test or endovascular treatment, and rSO_2_ began to improve from early phase after reflow [31, 34, 36]. No studies have been reported to evaluate intestinal viability after reperfusion by using the finger-mounted oximeter. Thus, we hypothesized that intestinal rSO_2_ reflects tissue oxygenation after reperfusion and can be used for the rapid assessment of intestinal viability.

The viable intestine showed rapid recovery of rSO_2_ in 10 min after declamping, and these animals survived for 1 week. In the viable intestine with intact microvessels, reperfusion causes rapid influx of oxyhemoglobin into intestinal tissue, leading to rSO_2_ increase. Conversely, rSO_2_ of the intestine with impaired microvessels did not increase after declamping because oxyhemoglobin could not flow into tissue. These findings implied the clinical usefulness of finger-mounted oximeter for the rapid intraoperative assessment of intestinal viability by measuring rSO_2_ shortly after releasing strangulation. The optimal time point for the human intestine should therefore be determined.

In addition, this device has potential applications for use in association with other ischemic diseases. The minimum horizontal measurement range of this oximeter is about 8 mm; the range form light-emitting diodes to photodiodes [6], suggesting the possibility that it could be applicable to the verification of narrow-range ischemia such as non-occlusive mesenteric ischemia or incarceration of Richter hernia.

The main limitation associated with this study is that this device has not been used to evaluate human intestine. The measurement depth of 4–5 mm was optimal for the rat intestine, but it is unclear whether this depth is suitable for the human intestine as well. New devices with variable depth have been developed for various types of tissue. For future clinical application, it is necessary to verify the optimal depth for the human intestine. In addition, the application for laparoscopic surgery is also expected. Unfortunately, it is not possible in the present shape, however, device upgrades such as improvement of probe structure or wireless data transmission might make it possible to use this device during laparoscopic surgery in the near future.

Another limitation is the brevity of follow up period after I/R injury. In this study, the postoperative period was only seven days and we did not evaluate the non-necrotizing irreversible variation such as post-ischemic bowel stricture [37, 38]. Further studies are needed to determine whether the assessment of rSO_2_ can also predicts long-term intestinal dysfunction.

## Conclusions

Real-time rSO_2_ monitoring using a finger-mounted tissue oximeter was useful in our animal model of I/R injury to assess intestinal ischemia and viability. If this device can be used clinically, then intraoperative decisions regarding intestinal preservation can be made rapidly and accurately, and unnecessary resection or postoperative intestinal necrosis may thus be avoided.
